# RETRACTION: Four Cases of Abdominal Expander Implantation in Adult Chronic Osteomyelitis of Lower Extremity With Soft Tissue Defect and Literature Review

**DOI:** 10.1111/iwj.70665

**Published:** 2025-04-23

## Abstract

**RETRACTION:** ZhangX. , ChenY. , WangG. , DingP. , YangX. , and ZhaoZ. , “Four Cases of Abdominal Expander Implantation in Adult Chronic Osteomyelitis of Lower Extremity With Soft Tissue Defect and Literature Review,” International Wound Journal 19, no. 8 (2022): 1980–1989, 10.1111/iwj.13798.35302286 PMC9705185

The above article, published online on 18 March 2022, in Wiley Online Library (http://onlinelibrary.wiley.com/), has been retracted by agreement between the journal Editor in Chief, Professor Keith Harding; and John Wiley & Sons Ltd. Following an investigation by the publisher, all parties have concluded that this article was accepted solely on the basis of a compromised peer review process. The editors have therefore decided to retract the article. The authors did not respond to our notice regarding the retraction.

**RETRACTION:** X. Zhang , Y. Chen , G. Wang , P. Ding , X. Yang , and Z. Zhao , “Four Cases of Abdominal Expander Implantation in Adult Chronic Osteomyelitis of Lower Extremity With Soft Tissue Defect and Literature Review,” International Wound Journal 19, no. 8 (2022): 1980–1989, 10.1111/iwj.13798.35302286 PMC9705185

The above article, published online on 18 March 2022, in Wiley Online Library (http://onlinelibrary.wiley.com/), has been retracted by agreement between the journal Editor in Chief, Professor Keith Harding; and John Wiley & Sons Ltd. Following an investigation by the publisher, all parties have concluded that this article was accepted solely on the basis of a compromised peer review process. The editors have therefore decided to retract the article. The authors did not respond to our notice regarding the retraction.

